# Long-term organic fertilization enhances paddy soil nitrogen availability through direct nutrient stimulation and complementary diazotroph-mediated pathway

**DOI:** 10.3389/fmicb.2026.1923757

**Published:** 2026-10-08

**Authors:** Li Wen, Chao Li, Kaikai Cheng, Lihong Shi, Lingling Liu, Shijie Qin, Ke Wang, Peng Jiang, Haiming Tang

**Affiliations:** 1 Hunan Cultivated Land and Agricultural Eco-Environment Institute, Changsha, Hunan, China; 2 Yuelushan Laboratory, Changsha, Hunan, China

**Keywords:** diazotrophic community, long-term organic fertilization, *nif*H gene, nitrogen availability, nitrogen fixation

## Abstract

**Introduction:**

Long-term organic fertilization is known to improve soil fertility, but the mechanisms by which it enhances soil nitrogen (N) availability, particularly the relative importance of direct nutrient stimulation versus diazotroph-mediated pathways, remain unclear.

**Methods:**

We examined the effects of four fertilization regimes (without fertilizer, CK; 100% chemical fertilizer, NPK; rice straw plus chemical fertilizer, RS; and 30% organic manure plus 70% chemical fertilizer, OM) on soil properties, diazotrophic community composition (*nif*H gene amplicon sequencing), N_2_ fixation activity (potential BNF and nitrogenase concentration), and soil N pools (NH_4_^+^–N, NO_3_^−^–N, and MBN) in a 37 year double-cropping paddy rice field experiment.

**Results and discussion:**

Long‑term organic fertilization, particularly OM, significantly increased soil organic carbon, total nitrogen and microelements (Mo, Fe, V and AMo). Relative to CK, OM treatment increased soil potential BNF rates by 342% and nitrogenase concentration by 178%, and maintained the highest soil inorganic nitrogen and MBN pools. Soil diazotrophic community composition (PCoA Axis1), rather than alpha diversity or *nif*H gene abundance, was the strongest correlate of potential BNF rates (*r* = 0.70, *p* < 0.001) and nitrogenase concentration (*r* = 0.85, *p* < 0.001). Partial least squares path modeling revealed that soil nutrients influenced soil N availability through dual pathways: a dominant direct effect on soil N fixation potential (0.734, *p* = 0.002), and a significant indirect effect mediated by soil diazotrophic community composition and N fixation activity (0.330). The model explained 98.8% of the variance in soil N availability with a goodness of fit of 0.842. In contrast, soil microelements showed negligible direct or indirect effects. These findings demonstrate that long-term organic fertilization enhances paddy soil N availability through coupled mechanisms: a dominant direct stimulation of N fixation by increased carbon and nutrient availability, complemented by a secondary pathway involving shifts in diazotrophic community composition and their concomitant N fixation activity. This study provides mechanistic evidence that sustaining soil N fertility in intensive rice agriculture depends on both adequate organic nutrient inputs and the maintenance of functional diazotrophic communities.

## 1 Introduction

Maintaining soil nitrogen (N hereafter) availability while reducing chemical fertilizer input is a central challenge for sustainable rice production in double cropping systems. In long-term intensively managed paddy fields, excessive reliance on synthetic N fertilizers has led to declining N use efficiency, yield stagnation, and adverse environmental consequences, including ammonia volatilization and nitrate leaching (Ladha et al., 2005; Chen et al., 2014; Lee, 2021). The incorporation of organic amendments, such as crop straw and livestock manure, has been widely adopted as an alternative strategy to sustain soil fertility and partially substitute for chemical N inputs (Tian et al., 2015; Li et al., 2023). Long-term organic fertilization not only increases soil organic carbon (SOC hereafter) and total N pools but also improves soil microbial activity, aggregate stability, and overall soil health in flooded rice ecosystems (Tong et al., 2014; Bian et al., 2024; Chen Y. L. et al., 2026). However, whether organic amendments enhance soil N availability primarily through direct nutrient supply or through indirect, biologically mediated pathways remains inadequately understood. Resolving this uncertainty is critical for optimizing organic amendment strategies in intensive agriculture.

Soil diazotrophic communities, which possess the nitrogenase enzyme capable of reducing atmospheric dinitrogen to ammonia, are increasingly recognized as key mediators of N availability and associated ecosystem functions in agricultural soils (Ray and Daniel, 2004; Reed et al., 2011). In double-cropping rice systems, repeated flooding and organic matter inputs create a unique habitat that supports diverse diazotrophs, ranging from obligate anaerobes to facultative and aerobic species (Hsu and Buckley, 2009; Wang Q. et al., 2021). A traditional ecological assumption posits that higher taxonomic diversity of a functional guild enhances its process rates through niche complementarity and functional redundancy (Wagg et al., 2014). However, emerging evidence from long-term fertilization experiments suggests that in paddy soils, community composition and the presence of functionally responsive taxa often exert stronger influences on biological nitrogen fixation (BNF hereafter) than overall taxonomic diversity (Liu et al., 2023). For example, continuous organic manure application tends to shift soil diazotrophic communities toward taxa with higher nitrogenase activity, whereas straw incorporation may promote distinct assemblages depending on carbon quality (Liao et al., 2018; Yang et al., 2021). Importantly, changes in soil diazotrophic community composition may not only affect BNF but also propagate through the N cascade to influence soil inorganic nitrogen pools (Philippot et al., 2024). Concurrently, the nitrogenase cofactor components molybdenum (Mo), iron (Fe), and vanadium (V) are essential structural constituents of nitrogenase cofactors, and are hypothesized to limit biological nitrogen fixation (BNF) in terrestrial ecosystems (Sheng et al., 2023; Butler et al., 2025), yet their importance under long-term organic fertilization in paddy soils remains unknown. Despite these advances, a critical knowledge gap persists regarding whether and how the diazotroph mediated N cascade operates: specifically, the relative importance of direct nutrient stimulation versus indirect community-mediated pathways, whether diazotrophic community composition or diversity predominantly drives the effect, whether the pathway is direct or indirect, and whether soil nutrients or microelements play the dominant role in mediating the soil BNF response to long-term organic fertilization. This gap limits our ability to predict the functional consequences of long-term organic amendment strategies in intensive rice agriculture.

We hypothesized that long-term organic fertilization enhances paddy soil nitrogen availability predominantly through an indirect, diazotroph-mediated N pathway, wherein organic inputs reshape diazotrophic community composition toward functionally responsive keystone taxa, which in turn stimulate N_2_ fixation activity and ultimately increase soil inorganic nitrogen pools, and that this biologically mediated pathway would operate alongside direct nutrient stimulation of nitrogen fixation. This hypothesis is grounded in the “mass ratio hypothesis” (Grime, 1998), which asserts that ecosystem functions are driven by dominant species traits rather than species richness, and is supported by evidence that specific diazotrophic taxa, rather than overall diversity, regulate BNF in paddy soils (Liu et al., 2023; Philippot et al., 2024). We further expected that this indirect pathway would dominate over direct nutrient effects, and that diazotrophic community composition would surpass diversity in driving BNF and N availability. Regarding soil microelements, we considered their role in this pathway to be minor relative to bulk soil nutrients, given that long-term organic inputs may have alleviated their potential limitation.

To test this hypothesis, we conducted a comprehensive investigation using a long-term field experiment in a double cropping rice system. We aimed to (1) determine whether the diazotroph-mediated effect on soil N availability is predominantly governed by community composition or diversity, and whether it operates through direct versus indirect pathways; (2) evaluate the contributions of bulk soil nutrients versus microelements in driving the observed responses, and (3) identify functionally responsive diazotrophic taxa enriched by organic fertilization that may underlie the enhanced N pathway. This study provides a mechanistic understanding of how long-term organic amendments sustain soil fertility in intensive rice agriculture.

## 2 Materials and methods

### 2.1 Soil site and experiment description

The long-term fertilization experimental site was established in 1986, located in Ningxiang County, Hunan Province (112°18′E, 28°07′N). The site has a subtropical monsoon climate, with mean annual temperature, evaporation and precipitation of 16.8 °C, 1353.9 mm and 1553.7 mm, respectively. For this field trial, a double rice cropping system was set up. The soil type was sandy loam paddy soils. The experiment consisted of four treatments, including a treatment without fertilizer (CK, as control), 100% chemical fertilizer (NPK), rice straw plus chemical fertilizer (RS), and 30% organic manure plus 70% chemical fertilizer (OM). The field experiment was set up a complete randomized block design consisting of three replicate plots (10.0 m × 6.67 m each plot). For early rice, the total fertilizer application rates across all fertilization treatments were nitrogen (N) 142.5 kg ha^−1^, phosphorus pentoxide (P₂O₅) 54.0 kg ha^−1^, and potassium sulfate (K₂O) 63.0 kg ha^−1^; for late rice, the total rates were N 157.5 kg ha^−1^, P₂O₅ 43.2 kg ha^−1^, and K₂O 81.0 kg ha^−1^. The straw return rate to the field was 2775.0 kg ha^−1^ in the early rice season (with straw containing 0.65% N, 0.13% P₂O₅, and 0.89% K₂O), and 3600.0 kg ha^−1^ in the late rice season (with straw containing 0.68% N, 0.15% P₂O₅, and 0.91% K₂O). For both early and late rice crops treated with organic fertilizer, the fertilizer used was well-decomposed chicken manure. In the early and later rice season, the application rates for treatments receiving 30% organic fertilizer were 2625.0 kg ha^−1^, 2670.0 kg ha^−1^, respectively (with organic fertilizer containing 1.77% N, 0.80% P₂O₅, and 1.12% K₂O). All treatments (except CK) were based on equivalent nitrogen levels, with any deficiencies in nitrogen, phosphorus, or potassium supplemented with chemical fertilizers. The total nitrogen, phosphorus and potassium inputs were the same for all fertilization treatments (except CK) during early rice and later rice during whole growth period. N and K₂O were applied as basal fertilizer and top dressing, respectively: the basal fertilizer is applied at plowing, while the top dressing is applied 7 days after transplanting, with a basal-to-top dressing ratio of 7:3. P₂O₅ is applied as a single basal fertilizer during plowing. The detail information on the fertilization patterns and field management has been described previously (Tang et al., 2018).

During the harvest of late rice in October 2023, soil samples were randomly collected from the 0–20 cm soil layer using five cores (diameter 6 cm) and were then thoroughly mixed. The visible crop roots and rock fragments were picked out after sieving using a 2 mm mesh. Soil samples were divided into three subsamples: one portion was air dried for physicochemical property analysis, a second portion was stored at 4 °C to determine the soil ammonium nitrogen (NH_4_^+^–N), nitrate nitrogen (NO_3_^−^–N), microbial biomass nitrogen (MBN), nitrogenase activity and potential BNF rates, and the remaining portion was stored at −80 °C until the subsequent microbial analysis.

### 2.2 Soil property and nitrogenase activity analysis

The air-dried soil samples were analysed for soil pH, soil organic carbon (SOC), total nitrogen (TN), total phosphorus (TP), molybdenum (Mo), available Mo (AMo), iron (Fe) and vanadium (V). The soil pH was determined using a pH meter in a 1:2.5 (weight:volume) soil-to-water ratio. SOC and TN were measured using a CN elementary analyzer. Molybdenum blue method was applied for measuring soil TP content. Soil microelement (Mo, Fe and V) contents were determined using inductively coupled plasma mass spectrometry (ICP-MS) after digestion with a mixture of HNO_3_, HClO_4_ and HF. Soil AMo was determined by ICP-MS by extraction with H_2_C_2_O_4_·2H_2_O–(NH_4_)_2_C_2_O_4_·H_2_O (LY/T 1121.9–2023). Soil ammonium nitrogen (NH₄^+^–N) and nitrate nitrogen (NO₃^−^–N) were extracted from 10 g of fresh soil with 100 mL of 2 mol L^−1^ KCl solution (soil to solution ratio of 1:10) by shaking at 200 rpm for 1 h at 25 °C (Hood-Nowotny et al., 2010). The extracts were filtered through Whatman No. 42 filter paper, and the concentrations of NH₄^+^–N and NO₃^−^–N in the filtrate were determined using a continuous flow analyzer (SEAL AutoAnalyzer 3, SEAL Analytical, Germany). Soil MBN content was determined by the chloroform fumigation extraction method using 0.5 mol L^−1^ K_2_SO_4_ as the extractant, and calculated using a conversion factor (kEN) of 0.45 (Brookes et al., 1985). The soil nitrogenase concentration was measured using a double antibody one-step sandwich enzyme-linked immunosorbent assay (ELISA) kit (Soil Nitrogenase ELISA Kit, Shanghai Enzyme linked Biotechnology Co., Ltd. Shanghai, China) and results were expressed as U/g dry soil. This assay quantifies the concentration of the dinitrogenase reductase protein (the *nif*H gene product) based on immunological reactivity. Soil C/N, C/P, and N/P ratios were calculated as mass ratios based on the measured SOC, TN, and TP contents (i.e., C/N = SOC/TN, C/P = SOC/TP, N/P = TN/TP).

### 2.3 Determination of soil potential biological nitrogen fixation rates

We determined soil potential BNF rates based on the acetylene reduction method in combination with a N_2_ uptake method (Li et al., 2018; Fang et al., 2024). Briefly, 10 g of soil was weighed and sealed in a 250 mL glass flask with rubber stopper upon sampling. Ten percent of the headspace of each flask was replaced with high-purity acetylene (cylinder acetylene with purity of 99.99%). Subsequently, the samples were incubated in the dark at 25 °C for 24 h. Then 20 mL of headspace gas was taken from the bottle using a stoppered syringe and transferred into a 12 mL pre-evacuated Labco headspace vial (Labco Exetainer, Labco Limited, UK), resulting in an overpressurized vial. This overpressurization was accounted for during subsequent gas chromatographic analysis by preparing calibration standards under the same overpressurized conditions. In addition, ethylene (C_2_H_4_) concentrations in acetylene only flasks and sample only flasks were estimated to ensure that the ethylene measured in the sample flasks was derived from nitrogenase enzyme activity. At the end of incubation, gas samples were determined using a gas chromatograph (Agilent GC 7890A, Agilent, USA) equipped with a flame ionization detector (FID). Acetylene reduction activity (nmol C_2_H_4_ g^−1^ dry mass day^−1^) was calculated from the ethylene concentration measured at the end of the 24 h incubation. The acetylene reduction activity was converted to N_2_ fixation rate (μg N kg^−1^ dry soil d^−1^) using a conversion factor of 1.37 for soil (Wang et al., 2019). All results are reported as potential nitrogen fixation rate measured under standard laboratory conditions.

### 2.4 DNA extraction, amplification, sequencing, and pre-analyses of sequencing data

DNA was isolated from 0.25 g soil using a FastDNA™ SPIN Kit for Soil (MP Biomedicals, Santa Ana, CA) according to the manufacturer’s instructions. The integrity of genomic DNA was detected through agarose gel electrophoresis, and the concentration and purity of genomic DNA were detected through the Nanodrop 2000 (10x Genomics, USA) and Qubit 3.0 Spectrophotometer (Thermo Fisher Scientific, USA). PCR amplifications of the nitrogenase reductase gene (*nif*H) of diazotrophs was carried out using the primer pair polF (TGCGAYCCSAARGCBGACTC)/polR (ATSGCCATCATYTCRCCGGA) (Poly et al., 2001). Amplicon sequencing was performed using the Illumina MiSeq platform at Genesky Biotechnologies Inc. (Shanghai, China).

### 2.5 Sequencing data analysis

We processed the raw high-throughput data using the Quantitative Insights Into Microbial Ecology (QIIME 2) toolkit and the UPARSE pipeline. DNA sequences were filtered based on quality files, and the remaining sequences were trimmed of barcodes and forward primers. In brief, the sequences of low quality (average score <20, length <100 bp) were excluded. Illumina MiSeq sequencing data were pre-processed to remove chimeras from the datasets. After optimizing the sequences, there were 1,030,007 high-quality sequences for diazotrophic genes. The UPARSE pipeline was used to generate an operational taxonomic unit (OTU) table. The diazotroph sequences were grouped into OTUs using a 97% identity threshold, and the most abundant sequence from each OTU was selected as a representative sequence for that OTU.

The diazotrophic gene sequences were converted to amino acid sequences and aligned with the diazotrophic gene database using the Framebot tool as described by Wang et al. (2013). Sequences with frameshift errors or internal stop codons were removed prior to downstream analysis. The number of sequences in each sample was rarefied to 120,000 reads for downstream analyses. All sequences were deposited in the NCBI Sequence Read Arch (SRA) database (accession numbers SUB16213260).

### 2.6 Statistical analysis

Prior to analysis, all data were assessed for normality and homogeneity, and natural logarithmic transformations were performed where necessary. One-way analysis of variance (ANOVA) followed by Tukey’s HSD test was performed using IBM SPSS Statistics (version 26.0, IBM Corp., Armonk, NY, USA) to determine significant differences in soil properties, N fixation parameters, and alpha diversity indices among the four fertilization treatments (*p* < 0.05). Alpha diversity indices, including the Shannon index, were calculated from the rarefied OTU table using QIIME 2 (version 2020.6). The relative abundance was determined by dividing the number of sequences affiliated with a given phylogenetic groups by the total number of sequences per sample. The Venn analysis was performed using the R package “VennDiagram” (version 1.6.20) to compare the microbial composition. Principal coordinate analysis (PCoA) based on Bray–Curtis distances was performed using the “vegan” package (version 2.6–4) in R (version 4.2.1, R Core Team, 2022) with rarefied OTU read counts (120,000 sequences per sample). Permutational multivariate analysis of variance (PERMANOVA, 999 permutations) was conducted using the adonis2 function in the “vegan” package to test for significant differences in diazotrophic community composition among treatments. The linear discriminant analysis (LDA) effect size (LEfSe) analysis was performed on the Wekemo Bioincloud online platform^3^ using default parameters, with an LDA score threshold of 2.5 and a significance level of *p* < 0.05. Spearman’s correlation analysis was performed using the “Hmisc” package (version 5.2–5) in R to evaluate the correlations among soil properties, diazotrophic community metrics, nitrogen fixation parameters and nitrogen availability. Linear regression analysis was performed and visualized using Sigmaplot (version 10.0, Systat Software Inc., San Jose, CA, USA) to evaluate relationships between diazotrophic community metrics (Shannon index, PCoA Axis1 and *nif*H gene abundance) and nitrogen fixation parameters (BNF and nitrogenase activity). The partial least squares path modeling (PLS-PM) analysis was performed using the “plspm” package in R to evaluate the multivariate effects on soil nitrogen availability. Five latent variables were constructed: bulk soil nutrients (SOC, TN, and TP), microelements (Mo, Fe, V and AMo), soil diazotrophic community (Shannon index, PCoA Axis1 and *nif*H gene abundance), nitrogen fixation function (nitrogenase concentration, potential BNF rate and MBN), and soil nitrogen availability (NH_4_^+^–N and NO_3_^−^–N). all latent variables were specified as reflective (mode A). Model performance was assessed using the goodness-of-fit (GoF) index and the coefficient of determination (*R*^2^). Direct, indirect, and cross-loading effects were also calculated to further interpret the relationships among latent variables.

## 3 Results

### 3.1 Soil properties, nitrogen fixation indexes and inorganic nitrogen pools under long-term fertilization regimes

Based on the one-way ANOVA analysis, long-term fertilization substantially altered soil physicochemical properties (Table 1). Compared with CK treatment, SOC, TN, TP, and total Mo, Fe and V contents were significantly increased in fertilization treatments and the highest values were observed in OM treatment (Table 1, *p* < 0.05). The C:N, C:P and N:P ratio was significantly lower under OM than under CK (Table 1, *p* < 0.05).

**Table 1:** Soil properties under four fertilization treatments (*n* = 3).

Soil properties	CK	NPK	RS	OM
pH	6.22 (0.19)a	6.33 (0.18)a	6.70 (0.20)a	6.82 (0.19)a
SOC (g kg^−1^)	17.88 (0.22)b	19.72 (0.80)b	22.49 (0.63)a	24.4 (0.50)a
TN (g kg^−1^)	1.83 (0.04)c	2.12 (0.04)c	2.50 (0.03)b	2.81 (0.04)a
TP (g kg^−1^)	0.43 (0.01)c	0.79 (0.02)b	0.74 (0.01)b	1.52 (0.06)a
Mo (mg kg^−1^)	0.44 (0.03)b	0.67 (0.05)a	0.68 (0.01)a	0.70 (0.03)a
Available Mo (mg kg^−1^)	0.02 (0.00)c	0.05 (0.01)bc	0.07 (0.00)b	0.12 (0.01)a
Fe (g kg^−1^)	26.87 (0.88)b	28.99 (0.19)a	27.76 (0.68)a	29.26(0.09)a
V (mg kg^−1^)	82.40 (1.81)b	89.09 (1.58)a	86.01 (0.83)ab	88.53 (0.31)a
C:N	9.33 (0.03)a	8.61 (0.10)b	9.55 (0.22)a	9.17 (0.14)a
C:P	41.53 (1.53)a	26.43 (1.02)b	32.50 (2.32)b	18.05 (0.41)c
N:P	4.45 (0.18)a	3.07 (0.15)b	3.40 (0.19)b	1.97 (0.07)c

Data are presented as mean ± standard error (SE). Different lowercase letters indicate significant difference among different fertilization treatments (*p* < 0.05). CK: without fertilization, NPK: only chemical fertilizer, RS: rice straw and chemical fertilizer, OM: 30% organic manure and 70% chemical fertilizer.

Fertilization significantly affected soil potential BNF rates and nitrogenase concentration (Figures 1a,b, *p* < 0.05). BNF rates significantly increased by 122.6 and 342.4% in RS and OM treatments compared with CK (Figure 1a, *p* < 0.05). However, the BNF rate in CK and NPK treatments showed no significant differences (Figure 1a, *p* > 0.05). Soil nitrogenase concentration was significantly enhanced relative to the CK after more than 35 years of fertilization, showing an order of OM > RS ≈ NPK > CK (Figure 1b, *p* < 0.05). Soil MBN content was significant higher in RS and OM treatments than in CK (Figure 1c, *p* < 0.05). Fertilization significantly increased both soil NH_4_^+^–N and NO_3_^−^–N contents, with a nearly 2.85–8.00 times increase compared with CK, and the highest value were observed in the OM treatment (Figures 1d,e, *p* < 0.05). Soil NH_4_^+^:NO_3_^−^ ratio remained largely unaffected by fertilization (Figure 1f, *p* > 0.05) (see Table 2).

**Figure 1:**
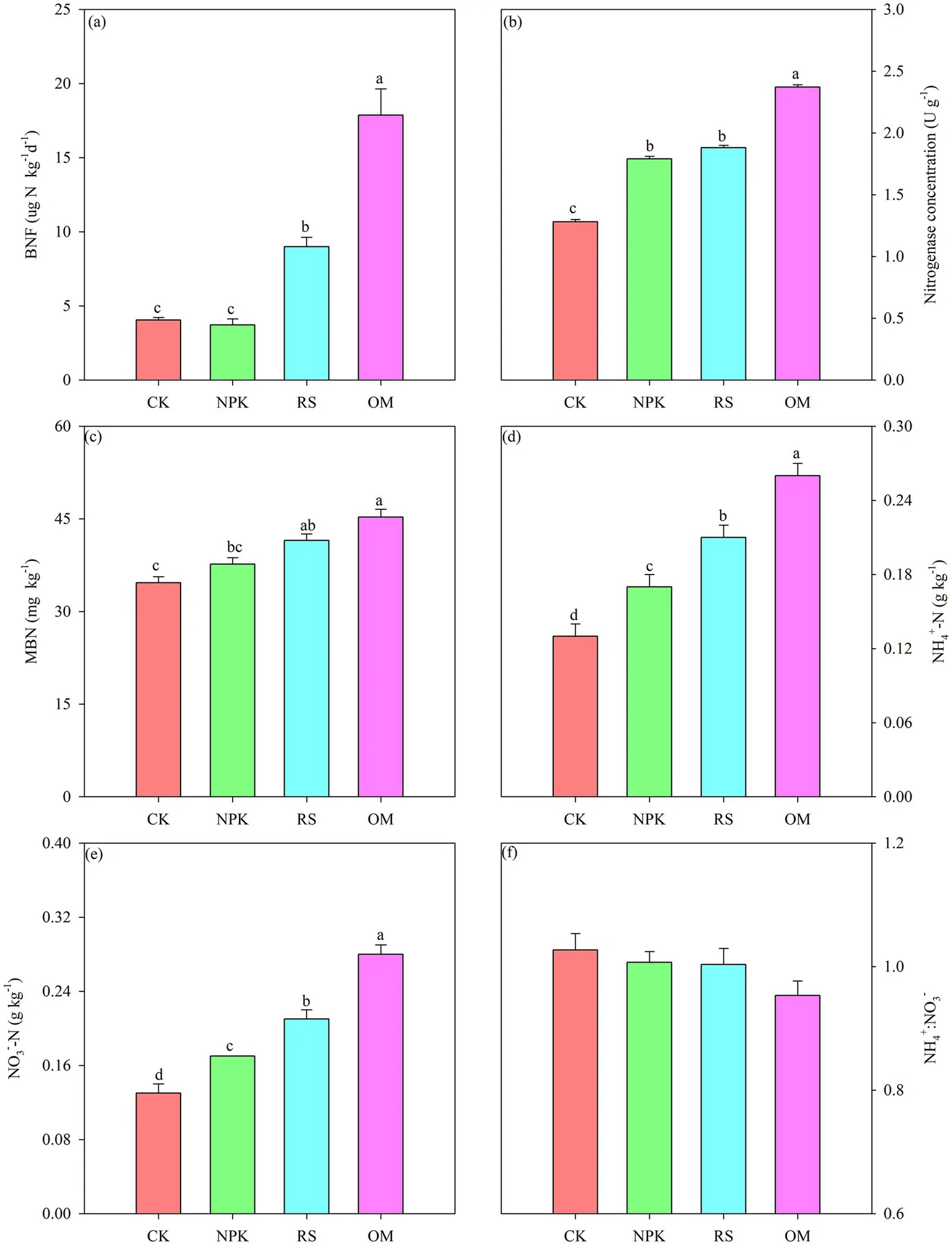
Soil potential biological nitrogen fixation rate (BNF) **(a)**, nitrogenase concentration **(b)**, soil microbial biomass nitrogen (MBN) **(c)**, soil ammonium nitrogen (NH_4_^+^–N) **(d)**, nitrate nitrogen (NO_3_^−^–N) **(e)**, and soil ammonium to nitrate nitrogen ratio (NH_4_^+^:NO_3_^−^) **(f)** under different fertilization treatments. Different lowercase letters denote significant differences at *p* < 0.05 (Tukey’s HSD test) between treatments. CK: Without fertilization; NPK: 100% chemical fertilizer; RS: Rice straw plus chemical fertilizer; OM: 30% organic manure plus 70% chemical fertilizer. Part of the raw data included in this figure were originally reported in our previous study (Guo et al., 2025, Acta Agriculturae Boreali-Sinica) and have been re-analyzed for this newly generated composite figure.

**Table 2:** Model quality indices and reliability of the partial least squares path model (PLS-PM).

Latent variable	MVs	C.alpha	AVE	*R* ^2^
Soil nutrients	3	0.964	0.934	-
Microelements	4	0.899	0.760	0.725
Diazotrophic community	3	0.704	0.647	0.724
Soil N fixation potential	3	0.955	0.918	0.962
Soil N availability	2	0.993	0.993	0.988
Goodness of fit				0.842

MVs, number of manifest variables; C.alpha, Cronbach’s alpha; AVE, average variance extracted.

Data are presented as mean ± standard error (SE). Different lowercase letters indicate significant difference among different fertilization treatments (*p* < 0.05). CK: without fertilization, NPK: only chemical fertilizer, RS: rice straw and chemical fertilizer, OM: 30% organic manure and 70% chemical fertilizer.

### 3.2 Shifts in soil diazotrophic community composition under long-term fertilization

A total of 2,579 OTUs were identified across all samples, with 155, 165, 180 and 253 unique OTUs in the CK, NPK, RS and OM treatment, respectively (Supplementary Figure S1). The Shannon index of diazotrophic community was significantly higher in all fertilized treatments than in CK, with no significant difference among NPK, RS and OM (Figure 2a). PCoA based on Bray–Curtis distance revealed clear separation of community structure among treatments, with the first two principal components explaining 67.88% of the total variation (PERMANOVA, *R*^2^ = 0.64, *p* < 0.01) (Figure 2b). Notably, the OM treatment formed a distinct cluster separate from CK (Figure 2b).

**Figure 2:**
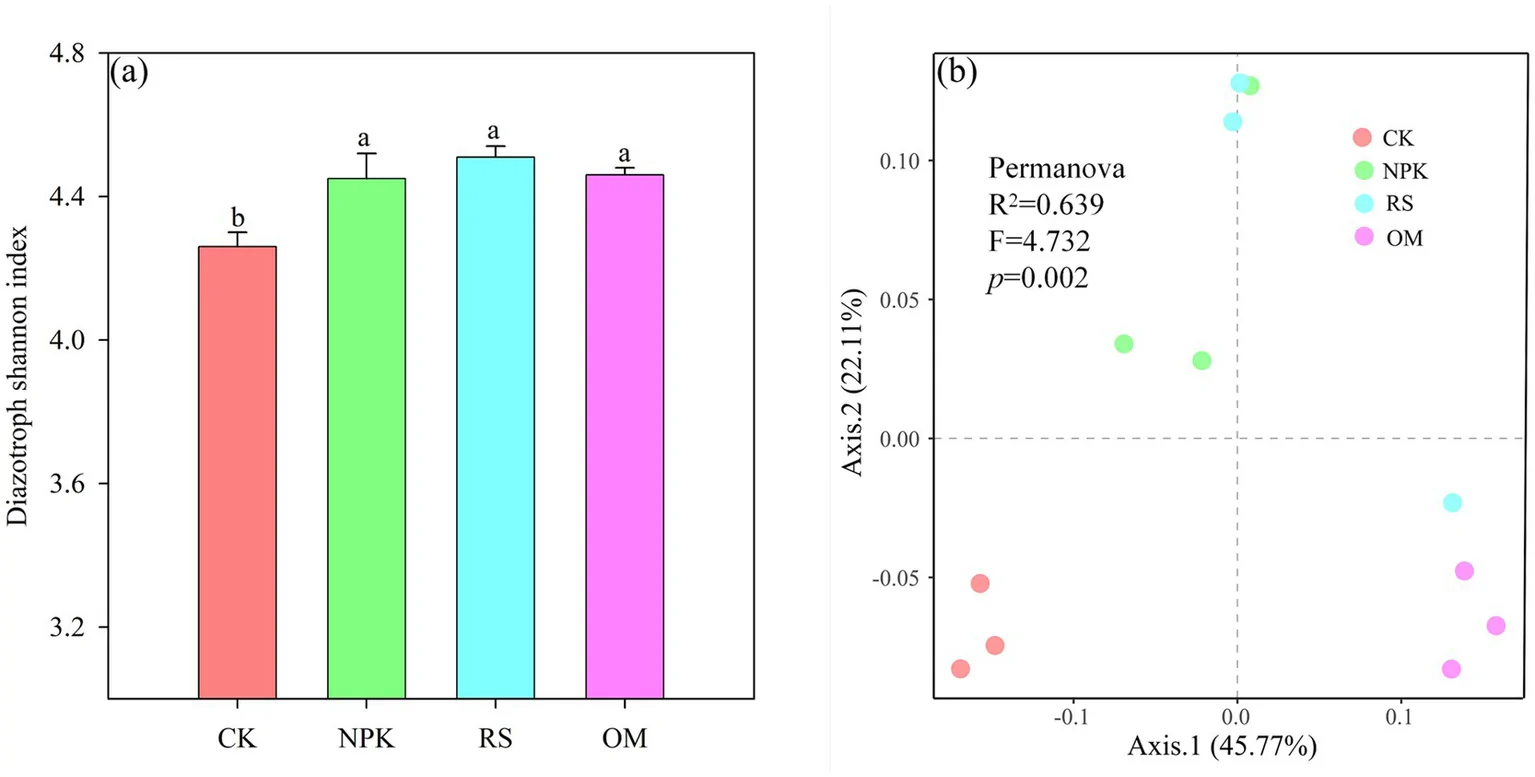
The Shannon index **(a)** and principal coordinate analysis (PCoA) results **(b)** of the diazotrophic community in each treatment. Different lowercase letters above the bars indicate significant differences at the *p* < 0.05 level (Tukey’s HSD test). CK: Without fertilization; NPK: 100% chemical fertilizer; RS: Rice straw plus chemical fertilizer; OM: 30% organic manure plus 70% chemical fertilizer.

At the phylum level, *Proteobacteria* dominated all communities (accounted for about 94.68%), with its highest relative abundance observed in OM and the lowest in CK (Supplementary Figur S2a, *p* < 0.05). At the genus level, *Bradyrhizobium*, *Azoarcus*, *Anaeromyxobacter*, *Pelobacter* and *Nitratireductor* collectively accounted for over 70% of the total diazotrophic community. Notably, *Bradyrhizobium* showed the highest relative abundance in OM soils, whereas *Azoarcus* and *Nitratireductor* were more abundant in CK (Supplementary Figure S2b, *p* < 0.05). LEfSe analysis identified 12 genera as biomarkers exclusively enriched in OM treatment (LDA > 2.5), predominantly belonging to the *Proteobacteria* (Supplementary Figure S3).

### 3.3 Relationships between soil diazotrophic community metrics, nitrogen fixation and nitrogen availability

The Shannon index exhibited no significant correlation with potential BNF rate (Figure 3a, *R*^2^ = 0.16, *p* > 0.05), but was positive correlated with nitrogenase concentration (Figure 3d, *R*^2^ = 0.40, *p* < 0.05). In contrast, soil diazotrophic community composition (PCoA Axis 1) exhibited strong positive correlations with both potential BNF rate (Figure 3b, *R*^2^ = 0.49, *p* < 0.001) and nitrogenase concentration (Figure 3e, *R*^2^ = 0.73, *p* < 0.001). Similarly, *nif*H abundance also correlated positively with both potential BNF rate (Figure 3c, *R*^2^ = 0.71, *p* < 0.001) and nitrogenase concentration (Figure 3f, *R*^2^ = 0.44, *p* < 0.05). Spearman correlation analysis revealed strong positive relationships between nitrogen availability, nitrogen fixation, and diazotrophic community (Supplementary Figure S4, *p* < 0.05).

**Figure 3:**
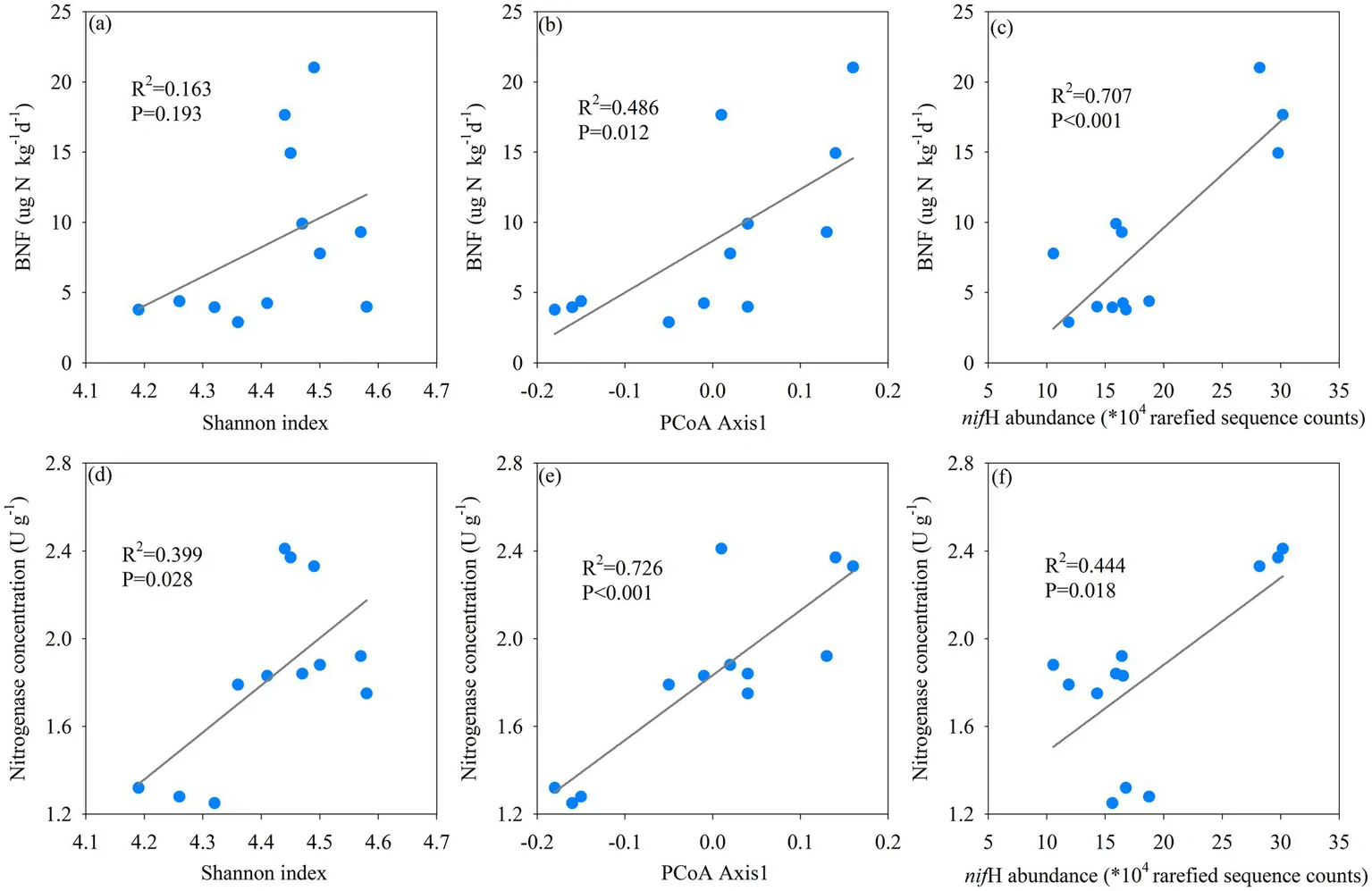
Relationships between diazotrophic community metrics (Shannon index, PCoA Axis1 and *nif*H gene abundance) and nitrogen fixation function (BNF and nitrogenase concentration). **(a)** BNF vs. Shannon index. **(b)** BNF vs. PCoA Axis 1. **(c)** BNF vs. *nif*H gene abundance. **(d)** Nitrogenase concentration vs. Shannon index. **(e)** Nitrogenase concentration vs. PCoA Axis 1. **(f)** Nitrogenase concentration vs. *nif*H gene abundance. Each point represents an individual sample (*n* = 12). Solid gray lines indicate linear regression fits. *R*^2^ and *p* values are shown in each panel.

PLS-PM was constructed to quantify the association between soil diazotrophic community, nitrogen fixation and nitrogen availability, and obtain a more comprehensive understanding of the direct and indirect effects of soil properties, diazotrophic communities, and nitrogen fixation on soil nitrogen availability (Figure 4). The model exhibited a good fit (GoF = 0.842) and explained 96.2% of the variation in nitrogen fixation potential and 98.8% of the variance in soil nitrogen availability (Table 2). Soil nutrients exerted a strong direct effect on microelement (0.852, *p* < 0.001) and diazotrophic community composition (0.755, *p* = 0.05), whereas soil microelements showed no significant direct effect (0.110, *p* > 0.05). Most importantly, soil nutrients directly enhanced soil nitrogen fixation potential (0.734, *p* = 0.002), and this direct path accounted for a larger proportion of the total effect than the indirect path mediated by diazotrophic community composition (0.330, *p* < 0.05). Soil nitrogen fixation potential in turn strongly affected soil nitrogen availability (0.904, *p* < 0.001). The total effect of soil nutrients on N availability was 0.954, comprising a non-significant direct effect (0.095, *p* = 0.438) and a dominant indirect effect transmitted through N fixation potential (0.859). Soil microelements showed negligible total effects on nitrogen fixation or nitrogen availability.

**Figure 4:**
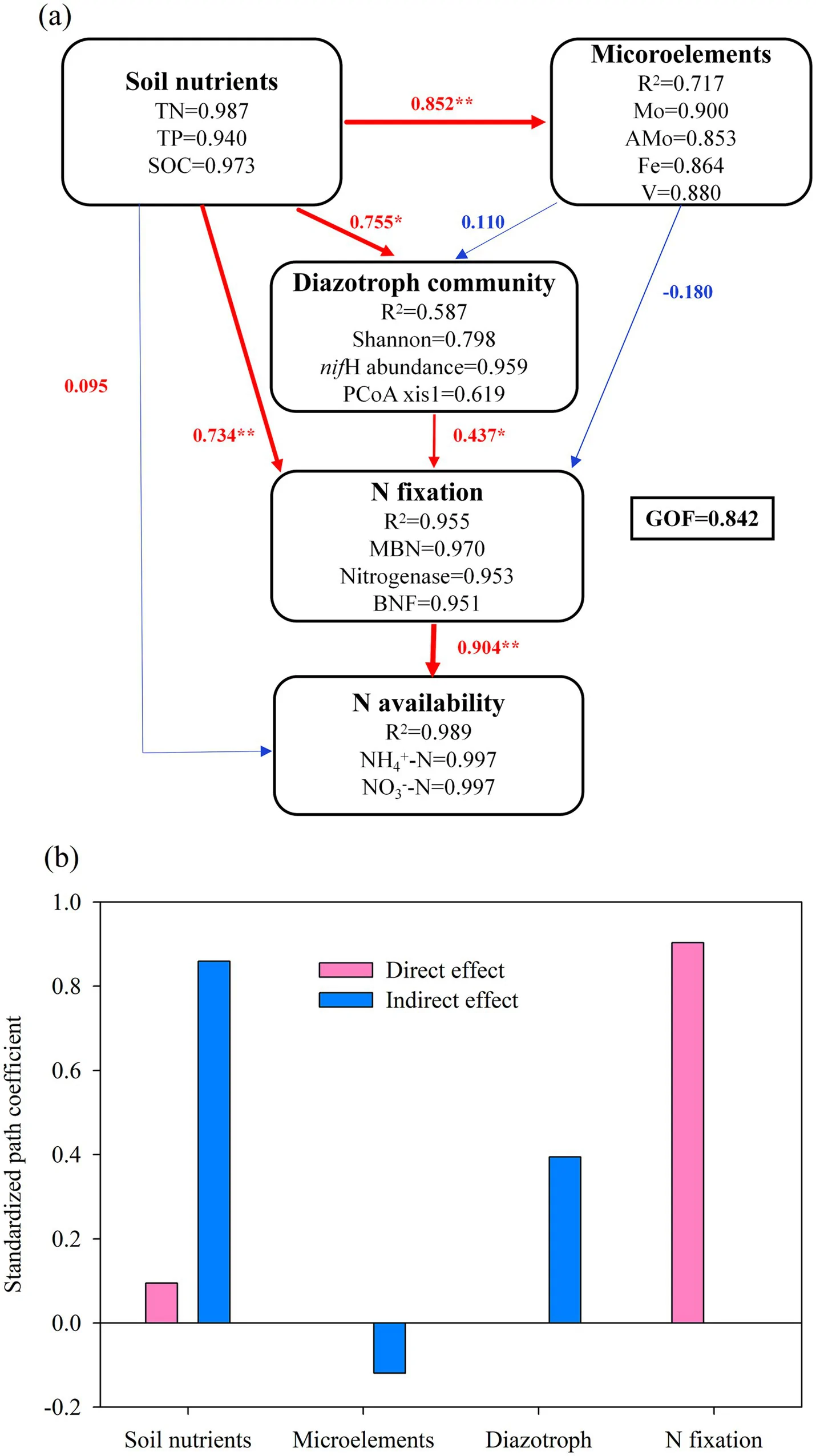
Partial least squares path model of the diazotroph-mediated pathway to soil nitrogen availability. **(a)** A partial least squares path model describing the effects of soil properties, diazotrophs, and nitrogen fixation function on the soil nitrogen availability. Latent variable of soil nutrients include SOC, TN, and TP. Latent variable of soil microelements includes V, Fe, Mo, and AMo. The latent variable for the diazotrophic community includes diazotrophs Shannon index, *PCoA* Axis1, and *nifH* gene abundance. The latent variable for soil nitrogen fixation includes potential BNF, nitrogenase concentration, and MBN. Path coefficients are labeled beside the arrow lines; numbers adjacent to arrows are standardized path coefficients. *R*^2^ values are shown within latent variables. **(b)** The standardized direct and indirect effects of soil nutrients, soil microelements, diazotrophic community, and nitrogen fixation function on nitrogen availability. CK: Without fertilization; NPK: 100% chemical fertilizer; RS: Rice straw plus chemical fertilizer; OM: 30% organic manure plus 70% chemical fertilizer.

## 4 Discussion

### 4.1 Long-term organic fertilization enhances soil nitrogen fixation and nitrogen availability through integrated nutrient provisioning

Our results demonstrate that long-term organic fertilization, particularly the combined application of organic and chemical fertilizer treatment (OM), significantly increased SOC, TN, TP, and the microelements Mo, Fe and V. Concomitantly, soil potential BNF rates, nitrogenase concentration and MBN were also markedly elevated (Figure 1, Table 1). These findings are consistent with previous studies showing that organic amendments improve soil fertility and microbial activity in paddy ecosystems (Liu et al., 2021; Bian et al., 2024; Gioacchini et al., 2024; Zhang et al., 2025). However, our study extends these observations by revealing a quantitatively suggestive linkage between enhanced BNF and subsequent increases in soil inorganic nitrogen pools, thus providing a statistic association consistent with a possible pathway from organic-derived energy inputs to plant-available nitrogen.

The markedly higher potential BNF rate and nitrogenase concentration under OM compared with NPK (Figures 1a,b) support the view that inorganic nitrogen inputs alone can suppress nitrogenase synthesis via feedback inhibition, whereas organic materials simultaneously provide carbon substrates that supply the ATP and reducing power essential for nitrogenase activity, and simultaneously alleviate nitrogen-induced repression (Tang et al., 2017). The enrichment of Mo, Fe, and V under OM treatment likely supplies essential cofactors for nitrogenase enzyme biosynthesis, further synergistically enhancing potential BNF rate (Sheng et al., 2023; Hu et al., 2024). Nevertheless, the magnitude of MBN increase (approximately 222% in OM relative to CK) was lower than that of potential BNF rate (approximately 342%), suggesting that a substantial portion of newly fixed nitrogen was allocated to the inorganic nitrogen pool (NH_4_^+^ and NO_3_^−^) rather than being entirely retained in microbial biomass (Shi et al., 2021). This partitioning may be ecologically advantageous, as inorganic nitrogen pools serve as readily available reservoirs for plant uptake or can be stabilized via soil colloids (Chen H. et al., 2026). This process, together with potential losses via denitrification or leaching in flooded paddy soils, still resulted in a net TN accumulation under OM (Figures 1d,e), indicating that gross BNF input sufficiently outpaced concurrent N effluxes. Long-term organic fertilization thus establishes a positive feedback loop wherein sufficient nutrients promote active nitrogen fixation, which in turn drives nitrogen accumulation, ultimately enhancing the multifunctionality of paddy soils (Wang Q. et al., 2021; Meng et al., 2023; Xu et al., 2024).

The differential responses between OM and RS treatments further corroborate the energy-limitation perspective: although both received equivalent total N inputs, the RS treatment enhanced BNF and nitrogen availability to a lesser degree than OM. This disparity likely reflects the differences in carbon quality and nutrient release patterns between straw and manure. Straw-derived carbon is dominated by recalcitrant cellulose and lignin, which decompose slowly and may limit energy supply for nitrogenase activity during the early growing season (Liao et al., 2018). In addition, the high C/N ratio of straw can intensify competition for mineral nitrogen between plants and diverse heterotrophic soil microorganisms (including both bacteria and fungi) during initial decomposition. This process temporarily immobilizes nitrogen in microbial biomass, partly offsetting the newly fixed input (Wang et al., 2023). In contrast, animal manure contains more labile organic carbon and a broader spectrum of nutrients, providing immediate energy sources for diazotrophs and accelerating the nitrogen cascade (Yang et al., 2021). Collectively, these findings suggest that the type and quality of organic amendments critically determine the magnitude of BNF enhancement and subsequent N availability in paddy systems.

### 4.2 Long-term organic fertilization enhances nitrogen fixation via community composition rather than alpha diversity

A central finding of this study is that soil diazotrophic community alpha diversity (Shannon index) showed no significant correlation with BNF rate, despite being significantly increased by long term organic fertilization (Figure 3a). In contrast, the first axis of PCoA, which captured the major gradient of community composition, was strongly positively correlated with potential BNF rate (Figure 3b, *p* < 0.05). Furthermore, *nif*H gene abundance was also significantly correlated with potential BNF rate (Figure 3c, *p* < 0.05). These results indicate that the strength of nitrogen fixation depends not on how many species are present or how evenly thay are distributed, but on which species dominate the community (Chen et al., 2024), a pattern consistent with the mass-ratio hypothesis, which proposes that dominant species exert the strongest influence on ecosystem processes (Grime, 1998).

The predominance of composition over alpha diversity can be attributed to substantial functional heterogeneity among diazotrophic taxa. Different genera are known to exhibit marked differences in carbon-source preference, tolerance to ammonium suppression, and other functional traits related to nitrogenase activity (Yang et al., 2021). In our study, long-term organic fertilization selectively enriched the relative abundance of certain diazotrophic genera, including *Bradyrhizobium* and *Geobacter*, while others such as *Azoarcus* and *Nitratireductor* declined (Supplementary Figure S2b). We note that this enrichment was based on relative abundance data and was not statistically confirmed by LEfSe analysis at the designated threshold (LDA score 2.5, Supplementary Figure S3). *Bradyrhizobium* has been reported in previous studies to efficiently couple carbon oxidation with nitrogen fixation under organic-rich conditions, and its enrichment under organic fertilization suggests a favorable response to increased carbon and nutrient availability (Dai et al., 2021). However, the specific functional contribution of these individual taxa to the observed increase in potential BNF in our system remains to be experimentally verified. Conversely, *Azoarcus* and *Nitratireductor*, which were more abundant in CK and NPK treatments, may possess different ecological strategies that are less conducive to high rates of nitrogen fixation under nutrient-limited conditions (Liao et al., 2018). This selective enrichment of functionally responsive diazotrophic taxa, rather than a general increase in alpha diversity, is therefore likely the key driver of enhanced nitrogen fixation potential.

Interestingly, the Shannon index exhibited a significant positive correlation with nitrogenase concentration but not with potential BNF rate. This divergence may reflect the different aspects of the nitrogen fixation process captured by each measurement. Nitrogenase concentration reflects the total abundance of the nitrogenase enzyme present in the soil microbial community, which may increase with greater community diversity through complementary resource use and functional redundancy (Wagg et al., 2014). In contrast, the potential BNF rate measured under standardized laboratory incubation represents the realized nitrogen fixation capacity under a specific set of conditions, which may depend more on the metabolic activity of specific competitively dominant taxa than on total enzyme abundance (Hsu and Buckley, 2009; Reed et al., 2011). This interpretation is consistent with the functional redundancy hypothesis, which posits that ecosystem functions are maintained by functionally equivalent species, but when environmental conditions change, the relative abundance of functionally distinct groups shifts, and overall species richness may not predict functional performance (Jordan, 2002). We note, however, that both measurements were conducted under standardized laboratory conditions, and the distinction between enzyme protein abundance and realized fixation rate under these conditions warrants further experimental validation. Collectively, these findings demonstrate that long-term organic fertilization enhances nitrogen fixation by restructuring the diazotrophic community toward enrichment specific taxa adapted to organic-rich conditions, rather than by simply increasing alpha diversity (Shi et al., 2021). Consequently, when assessing soil nitrogen fixation function, combining potential BNF rate with community composition analysis provides substantially more ecologically relevant insights than alpha diversity metrics or enzyme protein measurements alone.

### 4.3 Soil nutrients dominate the diazotroph-mediated pathway to nitrogen availability: implications and limitations

Elucidating the drivers of the diazotroph-mediated pathway from soil properties to soil inorganic nitrogen is critical for managing soil fertility in organic agricultural systems (Barbieri et al., 2023). Our results demonstrate that bulk soil nutrients (such as SOC, TN and TP), rather than microelements, serve as the primary controllers of this cascade. Specifically, bulk soil nutrients exerted dominant effects on soil diazotrophic community composition and nitrogen fixation, which in turn determined nitrogen availability, whereas microelements showed no significant direct or indirect effects. This aligns with the resource-based view that overall soil nutrient availability fundamentally governs the capacity of soil microbial communities to drive biogeochemical transformations (Yang et al., 2021). The substantial increases in soil carbon and nutrient pools following long-term organic amendment application likely provide the ATP and reducing power required to meet the high energetic cost of nitrogenase activity, thereby alleviating the energy constraints that limit BNF in nutrient-poor soils (Tang Y. et al., 2021). This interpretation is supported by empirical evidence that once baseline nutrient requirements are satisfied, energy availability, rather than microelement supply, becomes the predominant limiting factor for BNF (Wang J. et al., 2021; Zheng et al., 2023; Zhao et al., 2024; Butler et al., 2025). Collectively, the dominant effect of bulk soil nutrients over microelements observed in our study reinforces the emerging consensus that overall soil fertility, rather than individual micronutrients, governs soil diazotrophic community composition and nitrogen fixation function in intensively managed paddy ecosystem (Tang Y. et al., 2021; Yang et al., 2021).

Microelements, especially Mo and Fe, are essential structural constituents of the nitrogenase cofactor and have often been considered limiting for BNF in terrestrial ecosystems (Sheng et al., 2023; Hu et al., 2024; Butler et al., 2025). In this 37-year field experiment, long-term organic fertilization significantly increased soil Mo, Fe and V contents (Table 1); however, the PLS-PM analysis revealed that the direct effect of these soil microelements on diazotrophic communities was not significant. In our model, the pathway from microelements to nitrogen fixation was not specified as a direct link but was instead conceptualized as being mediated through the diazotrophic community, reflecting the established biochemical mechanism whereby microelements must first be assimilated by diazotrophs and incorporated into the nitrogenase cofactor before contributing to BNF (Seefeldt et al., 2009; Bellenger et al., 2020). This result indicates that microelements did not constitute a primary constraint on BNF in this paddy system. A parsimonious interpretation is that the indigenous concentrations of these cofactor metals may already meet the requirements for nitrogenase synthesis, rendering additional increments functionally redundant. The nitrogenase enzyme consists of two metalloprotein components (the Fe protein and the MoFe protein), each of which requires specific metal clusters for catalytic activity, and once baseline concentrations of these cofactor elements are sufficient for enzyme assembly, additional microelement inputs do not proportionally enhance nitrogen fixation activity (Sheng et al., 2023; Hu et al., 2024; Butler et al., 2025). Under such conditions of microelement sufficiency, the constraints on BNF would logically shift to other limiting resources. Consistent with this, the diazotrophic community composition and BNF potential in this system were governed predominantly by bulk soil nutrient status, as evidenced by the strong direct effect of soil nutrients on diazotrophic community composition (path coefficient = 0.755). This dominance of bulk soil nutrients likely reflects the substantial carbon and energy demand of nitrogen fixation: the nitrogenase reaction consumes 16ATP per N_2_ reduced (Ray and Daniel, 2004), and organic matter inputs alleviate these energetic constraints by supplying readily available carbon substrates, thereby promoting the proliferation and activity of copiotrophic diazotrophs. This interpretation is consistent with empirical evidence that BNF is not primarily constrained by P or Mo availability once baseline nutrient thresholds are surpassed (Wang J. et al., 2021; Zheng et al., 2023; Zhao et al., 2024; Butler et al., 2025), and with the emerging consensus that overall macronutrient fertility, rather than individual microelements, is the dominant driver of diazotrophic community assembly and nitrogen fixation function in intensively managed paddy ecosystem (Tang H. M. et al., 2021; Yang et al., 2021).

A particularly notable feature of the model is the partial mediation of the nutrient to nitrogen availability pathway by the biological component. Critically, this mediation is energetically driven, with soil carbon functioning primarily as an energy source to fuel nitrogenase, rather than as an independent fertility endpoint. The direct effect of soil nutrients on nitrogen availability was not significant, whereas the indirect effect transmitted through soil nitrogen fixation potential was dominant (0.859). Soil nutrients also exerted a significant direct effect on soil nitrogen fixation potential, indicating that enhanced carbon and nutrient availability directly stimulates nitrogen fixation in addition to the pathway mediated by the diazotrophic community. This finding underscores that the translation of improved soil fertility into plant-available nitrogen occurs primarily through both direct stimulation of microbial restructuring and nitrogen fixation, rather than through direct mineralization of organic nitrogen, at least within the causal framework of our model (Tang H. M. et al., 2021). Furthermore, the exceptionally high path coefficient from BNF to soil nitrogen availability (0.904, *R*^2^ = 0.939) quantitatively demonstrates that biological fixation is a major pathway sustaining the soil inorganic nitrogen pool under long-term organic fertilization. This provides strong evidence for the feasibility of relying on enhanced BNF to reduce or replace chemical N inputs in organic systems (Gao et al., 2024), although the extent of substitution may vary with management practices and environmental conditions. Collectively, these findings advance the traditional understanding of diazotroph-N relationships in three key ways. First, they identify community composition, not diversity or abundance, as the primary conduit through which nutrients affect BNF. Second, they provide quantitative support for the energy limitation hypothesis by demonstrating that showing organic carbon, rather than microelements cofactors, is the primary constraint on BNF in this paddy system. Third, they mechanistically explain why manure outperforms straw or chemical fertilizer alone by simultaneously supplying labile carbon to meet the high ATP demand of nitrogenase activity and enriching functionally responsive diazotrophic taxa that are adapted to organic -rich conditions. From an agronomic perspective, rational organic amendment strategies that optimize soil C, N, and P management are consequently more effective for enhancing BNF than approaches relying solely on microelement supplementation or single-strain microbial inoculants. Further research should investigate the minimum threshold of organic carbon input required to trigger diazotrophic community restructuring and subsequent enhancement of nitrogen fixation, as well as the long-term sustainability of this cascade under varying climatic and management conditions. Nonetheless, the prominent role of BNF identified in this study is inherently conditional upon the specific conditions of the 37-year paddy field experiment, including the type, quantity, and frequency of organic amendments, as well as the inherent baseline soil characteristics. The relative importance of BNF in relation to the direct mineralization of organic nitrogen may differ significantly in agricultural systems characterized by lower organic carbon inputs or different soil compositions, such as sandy or degraded soils. Furthermore, the fraction of soil inorganic nitrogen derived from BNF can exhibit significant temporal variability due to seasonal variations in temperature, moisture, and carbon accessibility. Therefore, future long-term studies covering a range of pedoclimatic zones are essential to confirm the universal applicability of this nitrogen cascade and to define the critical thresholds of organic carbon input, below which the restructuring of diazotrophic communities and the corresponding enhancement of BNF are ineffective.

## 5 Conclusion

This 37-year field experiment in a double-cropping paddy system demonstrates that long-term organic fertilization enhances soil nitrogen availability through coupled direct nutrient stimulation and diazotroph-mediated pathways. Among the four fertilization regimes, the combined organic and chemical fertilizer treatment (OM) significantly increased soil nutrients, potential biological nitrogen fixation (BNF) rates, nitrogenase concentration, and inorganic nitrogen pools. Soil diazotrophic community composition, rather than alpha diversity, emerged as the primary driver of soil nitrogen fixation, with functionally responsive taxa such as *Bradyrhizobium* being selectively enriched. PLS-PM modeling revealed that soil nutrients drove soil nitrogen availability through dual pathways: a dominant direct effect on soil nitrogen fixation potential and a complementary indirect effect mediated by diazotrophic community composition, with the final partial mediation model explaining 96.2% of the variance in soil nitrogen fixation potential and 98.8% of the variance in soil nitrogen availability. In contrast, soil microelements showed negligible direct or indirect effects under the conditions of this long-term study. These findings demonstrate that long-term organic fertilization enhance soil nitrogen availability primarily by increasing carbon and nutrient supply that directly stimulates nitrogen fixation, complemented by the restructuring of the diazotrophic community toward functionally responsive taxa. Our results provide mechanistic insights for designing organic amendment strategies to sustain soil fertility in intensive rice agriculture. Future long-term studies across diverse paddy soil types and pedoclimatic regions are needed to verify this pathway and to establish critical thresholds of organic carbon input for optimizing soil nitrogen supply in rice production.

## Data Availability

The raw data presented in the study are deposited in the NCBI repository, under accession numbers PRJNA1470345.

## Footnotes

3
https://www.bioincloud.tech

## Supplementary material

Supplementary Figure S3 The biomarkers of soil diazotrophic community of paddy soil.
