# Supplementary material for: Long-term organic fertilization enhances paddy soil nitrogen availability through direct nutrient stimulation and complementary diazotroph-mediated pathway
Source: Front Microbiol. 2026 Oct 8;17:1923757. doi: 10.3389/fmicb.2026.1923757 (PMC13687272; doi:10.3389/fmicb.2026.1923757)
Supplement: Supplementary file 1 [file Supplementary_File_1.DOC]

**Figure S1** The Venn of soil diazotrophic community under four fertilization treatments. CK, without fertilization; NPK, only chemical fertilizer; RS, rice straw and chemical fertilizer; OM, 30% organic manure and 70% chemical fertilizer. The same abbreviations are used below.

**Figure S2** The relative abundance of soil diazotrophic community at phyla (a) and gene (b) level.

**Figure S4** Spearman correlation between soil physicochemical properties, diazotrophic community metrics, nitrogen fixation index, and nitrogen availability. The significance levels of the predictors are **p* < 0.05. TN: total nitrogen; TP: total phosphorus; SOC: soil organic carbon; C:N: the ratio of SOC to TN; C:P: the ratio of SOC to TP; N:P: the ratio of TN to TP; AMo: available Mo; Shannon: the diazotrophic community shannon index; nifH: *nif*H gene abundance; MBN: soil microbial biomass nitrogen; Nitrogenase: soil nitrogenase concentration; Potential BNF: potential BNF rate; Ammonium: soil ammonium nitrogen; Nitrate: soil nitrate nitrogen; A:N: the ratio of ammonium to nitrate.
